# C-854-04. Evaluation of a Novel Decision Support System (BIRDS) Compared with Historic and Parkland-guided Resuscitation

**DOI:** 10.1093/jbcr/irag033.080

**Published:** 2026-04-06

**Authors:** Stacey Richerbach, Claudia Islas, Karen J Richey, Kevin N Foster

**Affiliations:** Diane & Bruce Halle Arizona Burn Center - Valleywise Health, Phoenix, AZ; Valleywise Health Medical Center, Phoenix, AZ; Diane & Bruce Halle Arizona Burn Center - Valleywise Health, Phoenix, AZ; Diane & Bruce Halle Arizona Burn Center - Valleywise Health, Phoenix, AZ

## Abstract

**Introduction:**

Fluid resuscitation in severe burns is complex, with risks of under- and over-resuscitation. Traditional formulas such as Parkland may not reflect actual physiological requirements. In 2023, our center incorporated a custom burn Clinical Decision Support Systems (CDSS) into our Electronic Health Record (EHR) to inform decisions regarding fluid resuscitation. The purpose of this project was to evaluate the novel CDSS, named the Burn Integrated Resuscitation Decision Support (BIRDS), against both historic institutional measures and the fluid recommendations yielded by the Parkland formula.

**Methods:**

Sixty-six consecutive patients (59 adults) supported by BIRDS were analyzed. Outcomes were compared with historical means using one-sample t-tests and with window-adjusted Parkland formula (PF4: 4 mL/kg/TBSA/24 h). Metrics included resuscitation duration, lactated Ringer’s (LR) delivery, urine output, and adherence to BIRDS hourly rate recommendations.

**Results:**

BIRDS patients had comparable TBSA to historical cohorts (40% vs 39%, *p*=.75) and weight (80 vs 86 kg, *p*=.08). Resuscitation duration was significantly shorter (23 h vs 31 h, *p*<10^-7^). Mean LR/kg/hr rate was markedly lower (4.78 vs 6.5 mL/kg/TBSA/hr, *p*<10^-12^), as was urine output (0.96 vs 2.3 mL/kg/hr, *p*<10^-20^). Compared with PF4, observed LR closely approximated expected totals (+369 mL, IQR −2.5 to +2.1 L); 49% of patients received >10% below PF4, 38% >10% above. Adults trended under PF4 in the first 8 h (−2.2 L) but over PF4 in hours 8–24 (+2.9 L, *p*<10^-10^). Adherence to BIRDS hourly recommendations was high (87%). Urine output achieved the fixed target range (0.35–0.65 mL/kg/hr) 20% of hours, with time in range decreasing as TBSA increased. Overall average urine output improved from 2.3 to 0.867 mL/kg/hr, *p*<.005.

**Conclusions:**

BIRDS-guided resuscitation significantly shortened duration and shifted practice away from historical high-rate resuscitation toward more physiologic, formula-adjacent volumes. While totals approximated Parkland overall, redistribution between early and late phases and difficulty achieving narrow urine output targets suggest areas for refinement. BIRDS demonstrates high adherence to rate recommendations, and reduced variability relative to historical practice, supporting its potential to modernize burn resuscitation.

**Applicability of Research to Practice:**

BIRDS offers a practical decision support tool that can be integrated into burn resuscitation workflows, fostering standardization, capacity for facility-driven customization, and alignment with evolving best practices in critical care.

**Funding for the Study:**

N/A.

